# Assessment of distraction callus in rabbits by monitoring of the electrical impedance of bone

**DOI:** 10.3109/17453674.2010.519910

**Published:** 2010-10-08

**Authors:** Takashi Yoshida, Wook-Cheol Kim, Yoshinobu Oka, Naotake Yamada, Toshikazu Kubo

**Affiliations:** Department of Orthopaedics, Graduate School of Medical Science, Kyoto Prefectural University of Medicine, Kyoto, Japan

## Abstract

**Background and purpose:**

Evaluation of distraction callus is important for determination of the optimal time for removal of external fixation. We attempted to determine whether there might be a relationship between the electrical impedance of bone and callus maturation, with a view to using impedance as a way of knowing when to remove a fixator.

**Methods:**

We applied an external lengthener to the right tibia of 24 rabbits and performed distraction at 1 mm/day for 10 days. Radiographs were taken and measurement of overall impedance between fixation pins was performed weekly after distraction. At weeks 2, 4, 6, and 8 after distraction (n = 6 each), resistivity of the bone to electrical conductivity was measured before killing. Cross-sectional area of the conduction pathway in callus and maximum bending stress were measured after excision of the tibia.

**Results:**

The overall impedance increased statistically significantly from 1 to 6 weeks after completion of distraction. The resistivity of bone decreased over 4 weeks and the cross-sectional area of callus decreased significantly over 6 weeks, while the maximum bending stress increased significantly over the same time. We observed a negative correlation between the cross-sectional area of callus and maximum bending stress.

**Interpretation:**

The impedance values, which are related to changes in electrical conductivity and the conduction pathway, increased due to the changes in the cross-sectional area of callus, despite the reduction in bone resistivity. Since the cross-sectional area of callus was correlated with maximum bending stress and the impedance values increased with the callus-remodeling process, we suggest that temporal increases in overall impedance reflect callus maturation.

Current clinical evaluation of distraction callus usually depends only on radiographic examinations, which may be unreliable; callus fracture or recurrence of deformity may occur after removal of the external fixation (Simpson and Kenwright 2000). Examples of methods previously reported for evaluation of distraction callus in animal studies include quantitative computed tomography (CT) (Harp et al. 1994, Reichel et al. 1998), dual-energy X-ray absorptiometry (DEXA) (Eyres et al. 1993, Reichel et al. 1998, Tselentakis et al. 2001), ultrasound (Eyres et al. 1993, Bail et al. 2002), and bone stiffness testing (Dwyer et al. 1996, Windhagen et al. 2000, Aarnes et al. 2006). CT (particularly with multiple examinations) is associated with additional radiation and is costly, and both CT and DEXA may be associated with artifacts attributable to external fixation. Ultrasound has problems with the accuracy and image quality of acoustic conduction. A bone-healing meter and bending testing still have various problems associated with the complexity of installing the measuring instruments and with the evaluation method.

Measurement of impedance is easy and useful for evaluation of conductive substances based on their electrical characteristics (Ohmine et al. 2004), and it is frequently used for evaluation of the physical properties and structure of substances in industry. It is also used for biological systems: for example, it has been used for measuring percentage of body fat and muscle volume of the extremities (Miyatani et al. 2001), as impedance increases with increase in body fat and muscle volume. Previous investigations of bone electrophysiology have shown the electrical and dielectric behavior of bone (Saha and Williams 1995), and the mechanical properties of human trabecular bone have been evaluated by electrical measurements (Sierpowska et al. 2005). We have previously reported increases in the electrical impedance of bone during fracture healing (Yoshida et al. 2009), focusing on both evaluation of bone and the fracture healing process. We investigated both bone conductivity and the conduction pathway itself, and we observed close involvement of the conduction pathway in the increase in impedance. Since steel pins are already inserted into long bones during callus distraction, simply by using these pins as electrodes, the overall impedance between pins can be measured without the need for any additional invasive procedures. We applied this method to distraction callus, which can be difficult to evaluate clinically. We analyzed the temporal changes in overall impedance values over time during the callus maturation process and investigated the relationship between changes in impedance values and the mechanical strength of the callus.

## Material and methods

This study was conducted according to the regulations of Kyoto Prefectural University of Medicine regarding animal research.

### Overall experimental design

A mid-diaphyseal osteotomy at the right tibia was performed in 24 Japanese white rabbits and an external fixator was applied. Radiographic examinations and measurement of overall impedance between fixation pins were performed once a week after osteotomy or completion of distraction. To analyze the electrical properties of distraction callus, bone resistivity as conductivity and callus cross-sectional area as conduction pathway were evaluated. A 3-point bending tester was used to measure the bending stress at the mid-callus. We examined temporal changes in the overall impedance, the electrical properties, and the mechanical strength of the distraction callus.

### Animals and surgical procedures

**Figure 1.**
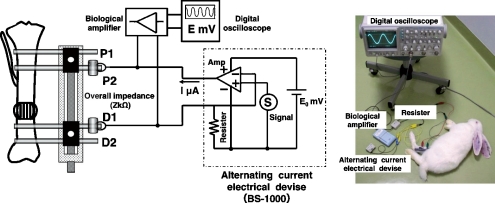
A. Diagram illustrating the experimental scheme. When measuring overall impedance, the electrical device was connected to the inner two pins (P2 and D1). The voltage (E, mV) between the electrodes was measured with a digital oscilloscope via a biological amplifier. The impedance values were calculated using the equation: Z (overall impedance) = E / I. B. Photograph showing measurement of overall impedance.

We used 24 five-week-old male Japanese white rabbits in this study (mean body weight: 1.1 (1.0–1.2) kg). Under intravenous anesthesia with pentobarbital at 30 mg/animal and local anesthesia with lidocaine at 5 mg/animal, 10-mm-long longitudinal skin incisions were made at 2 sites: one at one-third of the distance from the proximal end and the other at one-third of the distance from the distal end on the medial aspect of the right tibia. 2 threaded, stainless-steel pins (2.0-mm diameter, Kirschner wire with thread and trochar point, resistivity < 1 Ωm; Synthes, Inc, West Chester, PA) were inserted at each incision site perpendicular to the bone axis at an interval of 8 mm, followed by attachment of an Orthofix M-100 external fixator. Since anodic oxide coating was performed on the surface of the external fixator, the interval between the fixation pin and the clamp was in an insulated state. The 4 pins were designated P1, P2, D1, and D2 in order starting from the proximal end (Figure 1A). A 10-mm skin incision was made 5 mm proximal to the distal end pin (D1), the skin was peeled away to expose the periosteum, holes were drilled using a screw 2 mm in diameter, and osteotomy was performed with a bone chisel with a width of 1.2 mm. After 7 days, callus distraction was performed at a rate of 1 mm per day for 10 days.

The 24 animals were assigned to 4 groups (weeks 2, 4, 6, and 8 after distraction; n = 6 for each group). To analyze the electrical properties of bone during callus maturation, we measured bone resistivity for each group under intravenous anesthesia with pentobarbital, after removing the surrounding soft tissue including periosteum. During the removal of soft tissue, the posteromedial neurovascular bundle was preserved to maintain intramedullary blood flow. Hemostatic procedures were used when appropriate, at room temperature (22 ± 2°C) with a humidity of 50 ± 10%. For each animal, tissue removal and impedance value measurements were completed in about 20 min. The tibiae of the animals were excised after killing, followed by measurement of callus cross-sectional area to evaluate the conduction pathway and by measurement of maximum bending stress for determination of the mechanical properties of the distraction site.

### Radiographic examination

Radiographic examinations were performed under anesthesia after osteotomy and at 1-week intervals after completion of distraction. We obtained anteroposterior and mediolateral views with an effective voltage of 50 kV, a current of 100 mA, a focal distance of 100 cm, and an exposure time of 0.04 s.

### Measurement of overall impedance

The overall impedance (Z (kΩ)) between the P2 and D1 pins was measured using an alternating current (AC) device (MES Co. Ltd., Tokyo, Japan) (Kawamoto et al. 2005). The frequency was set at 2 ± 0.4 Hz to limit the reactance element of AC current to a negligible range during measurement. The impedance values were measured for each group under intravenous anesthesia before distraction and once a week after completion of distraction (Figure 1B). AC was applied using the device with a constant current output of 30 ± 6 μA and loading resistance of 0–60 kΩ. A 1-kΩ resistor was connected to the AC device. The output voltage (E_0_ (mV)) of the AC device was measured with a digital oscilloscope via a biological amplifier, and the constant current (I (μA)) was measured using the equation: I = E_0_/R_s_ (Figure 1A), where R_s_ is the standard resistance (1 kΩ). The voltage (E (mV)) between the electrodes was measured with a digital oscilloscope via a biological amplifier. The impedance values were calculated using the equation: Z (overall impedance) = E/I.

### Bone resistivity between P2 and D1

**Figure 2.**
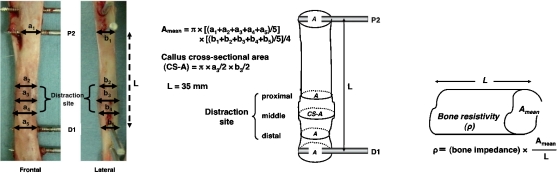
A. Photographs demonstrating measurement of bone resistivity between the P2 and D1 regions. Resistivity was calculated by measuring transverse diameter and anteroposterior diameter at the P2 region, the distraction site, and the D1 region, and bone tissue was assumed to be in the shape of an oval column based on averaging of these values. B. Diagrams illustrating measurement of mean cross-sectional area (A_mean_) and callus cross-sectional area (CS-A). C. Diagram illustrating measurement of bone resistivity (ρ).

The bone and distraction callus in the region from P2 to D1 were assumed to resemble a cylindrical conductor, and the bone resistivity (ρ (Ωm)) of each group was calculated as follows. The transverse diameter (a-values) and anteroposterior diameter (b-values) of the P2 region, proximal region of the distraction callus, middle of the distraction callus, distal region of the distraction callus, and D1 region were designated a_1_, a_2_, a_3_, a_4_, and a_5_ and b_1_, b_2_, b_3_, b_4_, and b_5_, respectively, and were measured using calipers. Their cross-sectional areas (A_mean_ (m^2^)) were calculated using the equation: A_mean_ = π × [(a_1_ + a_2_ + a_3_ + a_4_ + a_5_) / 5] × [(b_1_ + b_2_ +b_3_ + b_4_ + b_5_) / 5] / 4 (Figure 2A and B). The length between P2 and D1 (L (mm)) was constant at 35 mm for all animals after completion of distraction. The bone resistivity was calculated using the formula: ρ (bone resistivity) = (bone impedance) × A_mean_ / L (Figure 2C) using the bone impedance obtained in the state in which soft tissues, including periosteum, had been removed.

### Cross-sectional area of callus

The transverse diameter and anteroposterior diameter of the middle of the distraction callus of the excised bone tissue, which was the area enclosed by the callus envelope presented, were designated a_3_ and b_3_, and the cross section of the callus was approximated to resemble an oval shape, followed by calculation of the callus cross-sectional area (CS-A (mm^2^)) using the formula: CS-A (callus cross-sectional area) = π × a_3_/2 × b_3_/2 (Figure 2B).

### Maximum bending stress

The mechanical properties of the distraction callus for each group were measured using a static 3-point bending tester (Instron 5500R; Instron Corp, Canton, MA; load cell, 500 N; rupturing speed, 1 mm/min). In calculating the maximum bending stress (B_max_ (N/mm^2^)) of the distraction callus, we used a formula based on the assumption that the cross section of the distraction callus was oval. The distance between the fulcra at both ends was 40 mm. The bending stress was calculated from the formula: B_max_ (maximum bending stress) = M × y/I (Sharp 2003), where M is the moment, Force (N) × 20 (mm); y is the maximum distance from neutral axis, b_3_/2 (mm); and I is the inertia of the cross section, π × a_3_ × b_3_^3^/64 (mm^4^). Thus,


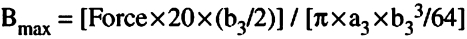


Statistical analysis was performed by testing among multiple groups using the one-way and repeated-measures ANOVA. Testing for the presence of a correlation was performed using Spearman's rank correlation coefficient.

## Results

### Radiographic findings

**Figure 3.**
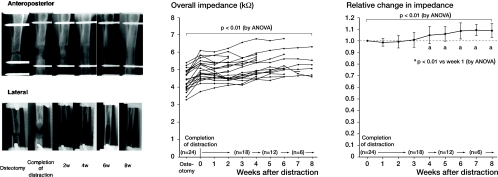
A. Anteroposterior and lateral radiographs revealing the formation of tubular structures of distraction callus at 4 weeks. Thickening of cortical bone and progression of the remodeling process were observed from 4 to 8 weeks. B. Graph showing sequential changes in overall impedance. The impedance values increased considerably, accompanying increases in the distance between fixation pins due to the 10-mm distraction. Although a slight decreasing trend was subsequently observed until 1 week, the impedance values increased over 6 weeks. C. Graph showing the rate of change based on the values at completion of distraction. Although a slight decreasing trend was observed over 1 week, the rate of change of impedance values increased substantially over 6 weeks and remained nearly constant at that time.

After completion of distraction, callus was observed near the proximal and distal ends of the osteotomized tibia, while radiolucent regions were observed in the center of distraction callus. These mineralization bands progressed toward the center and fused at 2 weeks. Corticalization and medullarization began from both the proximal end of the callus and the distal end, and then tubular formation by new cortical bone was observed at 4 weeks. Thickening of cortical bone and progression of the remodeling process were observed from 4 to 8 weeks (Figure 3A).

### Overall impedance

The overall impedance increased after completion of distraction, accompanied by an increase in length of 10 mm resulting from callus distraction. Although the values slightly decreased in week 1 after distraction, they increased with time after that and showed maximum values at weeks 6–8 (p < 0.01) (Figure 3B). Although the rate of change based on the impedance values at completion of distraction demonstrated a slight decreasing trend through week 1, it increased substantially from 1–6 weeks and remained nearly constant at that time (p < 0.01). Differences were observed between 1 week and 4, 5, 6, 7, and 8 weeks (p < 0.01) (Figure 3C). There was a positive correlation between the values of overall impedance and bone impedance obtained after removal of soft tissues including periosteum (p < 0.05, correlation coefficient 0.43).

### Bone resistivity

**Figure 4.**
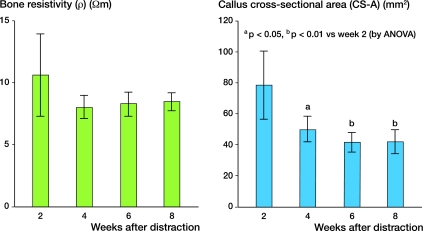

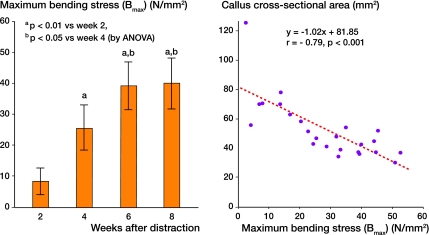
A. Graph showing changes in bone resistivity values. The values decreased substantially from 2 to 4 weeks after completion of distraction and subsequently remained nearly constant. B. Graph showing changes in callus cross-sectional area values. The values decreased over time during callus maturation, reaching a minimum at 6 weeks. Differences were observed between 2 weeks and 4, 6, and 8 weeks. C. Graph showing changes in maximum bending stress values. The values increased over time, and differences were observed between 2 weeks and 4, 6, and 8 weeks, and also between 4 weeks and 6 and 8 weeks. D. Graph showing correlation between callus cross-sectional area and maximum bending stress.

Although the values of bone resistivity decreased from weeks 2 to 4 after completion of distraction, they subsequently remained nearly constant over 8 weeks. No statistically significant differences were observed (Figure 4A).

### Cross-sectional area of callus

The callus cross-sectional area decreased over time, accompanying maturation of the distraction callus and reaching a minimum at 6 weeks after completion of distraction. Differences were observed between 2 weeks and 4, 6, and 8 weeks (p < 0.05, p < 0.01, and p < 0.01, respectively) (Figure 4B).

### Maximum bending stress

The maximum bending stress increased over time, accompanying maturation of the distraction callus. After having become nearly constant starting at 6 weeks, there were no differences observed between 6 and 8 weeks. However, differences were observed between 2 weeks and 4, 6, and 8 weeks (p < 0.01) and also between 4 weeks and 6 and 8 weeks (p < 0.05) (Figure 4C). There was a negative correlation between callus sectional area and maximum bending stress (p < 0.001; correlation coefficient −0.79) (Figure 4D).

## Discussion

Measurement of the overall impedance of distraction callus allows any changes in electrical properties to be evaluated during the callus maturation process. Advantages of this method include the absence of additional invasive procedures, since the pins already inserted for fixation are used as electrodes and measurements can be performed easily and frequently. Kawamoto et al. (2005) investigated enhancement of maturation of distraction callus and measured the impedance of excised bone. Hirashima et al. (2009) measured overall impedance in patients with a distal end fracture of the radius and they reported that impedance values increased concomitant with bone union. We also investigated the increases in overall impedance according to the fracture healing process and found that the values peaked at the time when mechanical strength reached a plateau (Yoshida et al. 2009). However, there have been no studies analyzing the changes in impedance values of distraction callus, and the reasons for the temporal increases in impedance values of bone with intramedullary blood flow still remain unknown. Weast and Astle (1981) measured the resistance rate in representative tissues to be 30,000 Ωm in dry bone and 30 Ωm in cortical bone moistened with physiological saline. Thus, the intramedullary blood flow is important for evaluation of the electrical conductivity of the callus.

One of the objectives of our study was to identify the factor elevating the overall impedance with callus maturation. The electrical properties of a substance are defined in terms of the distance between the substance, the electrical conductivity of the material, and the conduction pathway. Black and Mattson (1982) and Kosterich et al. (1984) have evaluated increases in electrical conductivity of bone associated with changes in bone maturation, such as corticalization and medullarization, in vitro. We investigated corticalization and medullarization as changes in bone resistivity in vivo. We also evaluated the current conduction pathway by investigating osteomorphological changes in the cross-sectional area of the distracted region. The measurement distance between P2 and D1 remained constant at 35 mm after completion of distraction, while the bone resistivity and the cross-sectional area of the distraction callus fluctuated during callus maturation. The bone resistivity decreased until 4 weeks after the completion of distraction, which influenced the bone by reducing the impedance. As a factor elevating the impedance, reduction of callus cross-sectional area, i.e. reduction of the conduction pathway, was substantially involved, as previously observed in a rabbit fracture model (Yoshida et al. 2009), and a strong negative correlation was noted between the callus sectional area and maximum bending stress. This shows that the reduction of callus cross-sectional area influences the elevation of impedance as the mechanical strength increases. We also considered that increases in the mechanical strength and impedance are involved through changes in the cross-sectional area of callus. We found that increases in the impedance disappear as changes in the callus cross-sectional area disappear. The process reaches bone maturation at this time point, which was also supported by the mechanical test findings. However, the impedance values would be influenced by the pathway of the current, and maximum bending stress is influenced by the trabecular structure, such as corticalization. The values of overall impedance are changed by all the factors associated with changes in bone structure and morphology during bone maturation.

Regarding the limitations of this study, detailed verification of the bone maturation, structure, and morphology by histological investigation and μCT remains to be performed, and we are planning to do this in the future.

We consider that bone maturation should be evaluated in individual cases based on the time course of changes in overall impedance, and not the absolute value. Our data suggest that measurement of the temporal changes in impedance values over time may be useful for evaluation of callus maturation.

## References

[CIT0001] Aarnes GT, Steen H, Kristiansen LP, Festo E, Ludvigsen P (2006). Optimum loading mode for axial stiffness testing in limb lengthening. J Orthop Res.

[CIT0002] Bail HJ, Kolbeck S, Krummrey G, Weiler A, Windhagen HJ, Hennies K, Raun K, Raschke MJ (2002). Ultrasound can predict regenerate stiffness in distraction osteogenesis. Clin Orthop.

[CIT0003] Black J, Mattson RU (1982). Relationship between porosity and mineralization in the haversian osteon. Calcif Tissue Int.

[CIT0004] Dwyer JS, Owen PJ, Evans GA, Kuiper JH, Reichardson JB (1996). Stiffness measurements to assess healing during leg lengthening: a preliminary report. J Bone Joint Surg (Br).

[CIT0005] Eyres KS, Bell MJ, Kanis JA (1993). Methods of assessing new bone formation during limb lengthening: ultrasonography, dual energy X-ray absorptiometry and radiography compared. J Bone Joint Surg (Br).

[CIT0006] Harp JH, Aronson J, Hollis M (1994). Noninvasive determination of bone stiffness for distraction osteogenesis by quantitative computed tomography scans. Clin Orthop.

[CIT0007] Hirashima T, Kim WC, Kawamoto K, Tsuchida Y, Oka Y, Hosokawa M, Yoshida T, Tsuji Y, Kudo T (2009). Evaluating of bone union of distal radius fracture by measuring impedance values. Orthopedics.

[CIT0008] Kawamoto K, Kim WC, Tsuchida Y, Tsuji Y, Fujioka M, Horii M, Mikami Y, Tokunaga D, Kubo T (2005). Effects of alternating current electrical stimulation on lengthening callus. J Pediatr Orthop B.

[CIT0009] Kosterich JD, Foster KR, Pollack SR (1984). Dielectric properties of fluid saturated bone—the effect of variation in conductivity of immersion fluid. IEEE Trans Biomed Eng.

[CIT0010] Miyatani M, Kanehisa H, Masuo Y, Ito M, Fukunaga T (2001). Validity of estimating limb muscle volume by bioelectrical impedance. J Appl Physiol.

[CIT0011] Ohmine Y, Morimoto T, Kinouchi Y, Iritani T, Takeuchi M, Haku M, Nishitani H (2004). Basic study of new diagnostic modality according to non-invasive measurement of the electrical conductivity of tissues. J Med Invest.

[CIT0012] Reichel H, Lebek S, Alter C, Hein W (1998). Biomechanical and densitometric bone properties after callus distraction in sheep. Clin Orthop.

[CIT0013] Saha S, Williams PA (1995). Comparison of the electrical and dielectric behavior of wet human cortical and cancellous bone tissue from the distal tibia. J Orthop Res.

[CIT0014] Sharp A (2003). Bicycles & Tricycles: A Classic Treatise on Their Design and Construction.

[CIT0015] Sierpowska J, Hakulinen MA, Töyräs J, Day JS, Weinans H, Jurvelin JS, Lappalainen R (2005). Prediction of mechanical properties of human trabecular bone by electrical measurements. Physiol Meas.

[CIT0016] Simpson AH, Kenwright J (2000). Fracture after distraction osteogenesis. J Bone Joint Surg (Br).

[CIT0017] Tselentakis G, Owen PJ, Richardson JB, Kuiper JH, Haddaway MJ, Dwyer JS, Evans GA (2001). Fracture stiffness in callotasis determined by dual-energy x-ray absorptiometry scanning. J Pediatr Orthop B.

[CIT0018] Weast RC, Astle MJ (1981). CRC handbook of chemistry and physics.

[CIT0019] Windhagen H, Kolbeck S, Bail H, Schmeling A, Raschke M (2000). Quantitative assessment of in vivo bone regeneration consolidation in distraction osteogenesis. J Orthop Res.

[CIT0020] Yoshida T, Kim WC, Kawamoto K, Hirashima T, Oka Y, Kubo T (2009). Measurement of bone electrical impedance in fracture healing. J Orthop Sci.

